# Functional neurological disorder: characterization, diagnosis, and outcome predictors in a Brazilian cohort

**DOI:** 10.1055/s-0046-1827064

**Published:** 2026-08-13

**Authors:** Lucas D'Andréa, Antonio Asciutti, Jordana Borges Camargo Diniz, Eduardo Genaro Mutarelli, Bruna Bartorelli

**Affiliations:** 1 Universidade de São Paulo, Faculdade de Medicina, Departamento de Psiquiatria, São Paulo SP, Brazil.; 2 Universidade de São Paulo, Faculdade de Medicina, Departamento de Neurologia, São Paulo SP, Brazil.; 3 Instituto de Neurologia de Goiânia, Departamento de Neurologia, Goiânia GO, Brazil.

**Keywords:** Conversion Disorder, Seizures, Psychotherapy, Group, Prognosis, Brazil

## Abstract

**Background:**

Functional neurological disorder (FND) is a prevalent, disabling neuropsychiatric condition recognized as a distinct clinical entity characterized by positive neurological signs. Despite growing international research, Brazilian data on this disoder remain scarce and predictors of clinical outcomes are poorly established.

**Objective:**

To describe the clinical profile of patients with FND in a Brazilian outpatient cohort and investigate predictors of functional improvement.

**Methods:**

We retrospectively analyzed medical records of 29 patients diagnosed with FND at the Somatic Disorders Outpatient Unit (SOMA) of the Institute of Psychiatry of Hospital das Clínicas, Universidade de São Paulo (IPq-HCFMUSP). Psychiatric diagnoses were established by board-certified psychiatrists using Diagnostic and Statistical Manual of Mental Disorders 5 ^th^ edition (DSM-5) criteria and structured clinical interview. Clinical features, psychiatric comorbidities, and therapeutic interventions were recorded. A multivariable Firth's bias-reduced logistic regression identified predictors of functional improvement.

**Results:**

The cohort included predominantly cisgender women (75.9%), with a mean age of 33.6 ± 13 years. The most common presentations were pain (51.7%) and paresis (48.3%); others included psychogenic nonepileptic seizures (PNES), sensory disturbances, and speech alterations. The most frequent positive neurological sign was Hoover's sign (20.7%). Psychiatric comorbidities included mood (44.8%), anxiety (20.7%), and personality disorders (10.3%). Overall, 31% achieved functional improvement. Group psychotherapy with a psychodynamic orientation significantly increased odds of improvement ( *p* = 0.036); eutony also showed a positive association (odds ratio [OR] = 5.68; *p* < 0.05). Absence of mood disorders was associated with lower odds of improvement (OR = 0.36; 95% confidence interval: 0.11–0.99; *p* = 0.048).

**Conclusion:**

The present is the first Brazilian study to employ multivariable modeling to identify predictors of functional outcomes in FND. Group psychotherapy and eutony were associated with improvement, reinforcing the value of multidisciplinary approaches and highlighting the importance of Latin American clinical data.

## INTRODUCTION

Functional neurological disorder (FND) is a prevalent, costly, and potentially disabling condition, characterized by neurological symptoms that cannot be explained by a structural neurological disease or other medical condition, but which present positive clinical signs that allow diagnosis. 1 2 It is estimated that FND accounts for 5 to 10% of outpatient neurology consultations and up to 16% of admissions to specialized services. 3 Historically, this disorder has been the target of stigma, often described as “hysteria” or “psychogenic disorder”, terms now considered inadequate as they reinforce exclusively psychological explanations and perpetuate the mind–body dualism. 4

The clinical and social impact of FND is considerable: patients frequently present with functional disability, high use of health services, and substantial socioeconomic costs. 4 For these reasons, the condition has been labeled the “silent epidemic of medicine” and a central challenge for both neurologists and psychiatrists. 5 6

An important milestone was the publication of the Diagnostic and Statistical Manual of Mental Disorders 5 ^th^ edition (DSM-5), which established diagnostic criteria based on a positive “rule-in” approach, emphasizing characteristic clinical signs (such as Hoover's sign, distractibility of tremors, and motor incongruence), in contrast to the previous logic of exclusion. 7 This change represented a significant advance in the destigmatization of FND, by providing a more integrated explanation centered on dysfunctional neurobiological networks. 8 9

From a pathophysiological perspective, studies using functional neuroimaging and brain connectivity show that FND is associated with dysfunction in networks responsible for attention, motor agency, interoceptive perception, and emotional regulation. 10 11 These findings support a biopsychosocial model, in which predisposing factors (trauma, genetic vulnerability), precipitating factors (acute stress, medical events), and perpetuating factors (avoidance, illness beliefs, social reinforcement) interact to maintain symptoms. 12

Clinical manifestations are heterogeneous, encompassing motor symptoms (weakness, tremor, dystonia, gait disorders, and movement disorders), psychogenic nonepileptic seizures (PNES), sensory disturbances, speech and voice alterations, and autonomic symptoms. 2 13 Not all subtypes were represented in our sample: patients with isolated PNES, sensory disturbances, or autonomic symptoms were referred to other specialized services and, therefore, are not included in this cohort. This broad spectrum reinforces the need for a specialized and careful diagnostic approach, based on positive signs and clear clinical communication, which are fundamental for treatment adherence. 14

In terms of treatment, there is consensus that a multidisciplinary approach is essential, combining diagnostic education, specific physiotherapy, psychotherapy (particularly cognitive-behavioral), occupational therapy, and adjunctive interventions. 6 15 16 17 Randomized clinical trials and consensus studies have shown consistent benefits of targeted physiotherapy for motor symptoms 16 and psychotherapy for nonepileptic seizures. 17

Despite these advances, the prognosis of FND remains unfavorable in many cases: systematic reviews indicate that most patients remain stable or worsen over time. 18 Favorable prognostic factors include early diagnosis, clear communication, and rapid initiation of treatment. Meanwhile, older age, psychiatric comorbidities, and delayed diagnosis are associated with poorer outcomes. 19

The present study aims to describe the clinical, diagnostic, and therapeutic characteristics of FND, reviewing current literature, and to analyze data from a Brazilian outpatient cohort of patients under treatment at the Institute of Psychiatry of Hospital das Clínicas, from Universidade de São Paulo's Medical School (IPq-HCFMUSP), São Paulo, Brazil, with emphasis on clinical predictors and factors associated with functional improvement.

## METHODS

The IPq-HCFMUSP is a high-complexity psychiatric hospital in São Paulo, where patients receive multidisciplinary treatment in in- and outpatient units. Patients with FND are referred to the Somatic Disorders Outpatient Unit (SOMA) after diagnostic assessment in specialized neurology wards, emergency units, or outpatient neurology services. Upon referral, all patients underwent a structured neurological and psychiatric evaluation.

Neurological diagnosis was established by a neurologist based on positive clinical signs according to DSM-5 criteria. 7 Psychiatric diagnoses were established by board-certified psychiatrists using these same criteria, supported by structured clinical interview (structured clinical interview for DSM-5 [SCID-5] or equivalent clinical evaluation).

Treatment allocation was not randomized; instead, it followed a standardized protocol based on clinical indication: group psychodynamic psychotherapy was offered to all patients who met criteria for group participation (absence of active psychotic symptoms or severe cognitive impairment); eutony was offered as a complementary body-oriented intervention to patients without significant physical contraindications; physiotherapy was indicated for those with predominant motor symptoms; individual psychotherapy was reserved for patients requiring individualized psychological work; and acupuncture was offered as an adjunctive intervention based on patient preference and clinical judgment.

Group psychodynamic psychotherapy was conducted weekly in 50-minute sessions with two psychologists. Eutony is a mind-body practice under the field of somatic education, which promotes the learning of tonic regulation through self-touch exercises, guided verbally by a eutonist. Sessions were also conducted weekly for 50 minutes. Patients could receive more than one treatment modality simultaneously during follow-up; in the statistical analysis, each modality was treated as an independent binary variable.

Data were collected from medical records of FND patients under treatment or discharged from follow-up in SOMA, with a data collection cutoff of March 2024. The epidemiologic characteristics were gathered and organized by a multidisciplinary team of psychologists, psychiatrists, and neurologists. Functional improvement was assessed based on the clinical evaluations of attending psychiatrists, as documented in the medical records. The relevant domains for this clinician-reported outcome included: self-care capacity, independence in performing activities of daily living (ADLs), level of autonomy, patient-endorsed subjective improvement, frequency of physical activity, engagement in community-based activities, and the ability to articulate the relationship between emotional states and neurological symptoms.

### Statistical analysis

Data are described as mean ± standard deviation (SD), median (interquartile range [IQR]), or count (%), as appropriate. Hypothesis testing was performed using the Fisher's exact, Student's T, or Chi-squared tests, as applicable. Firth's bias-reduced logistic regression was modeled to evaluate the relationship between possible predictors and functional improvement as the outcome of interest. Analyses were conducted using R (R Foundation for Statistical Computing).

## RESULTS

### Population characteristics

**Figure 1:**
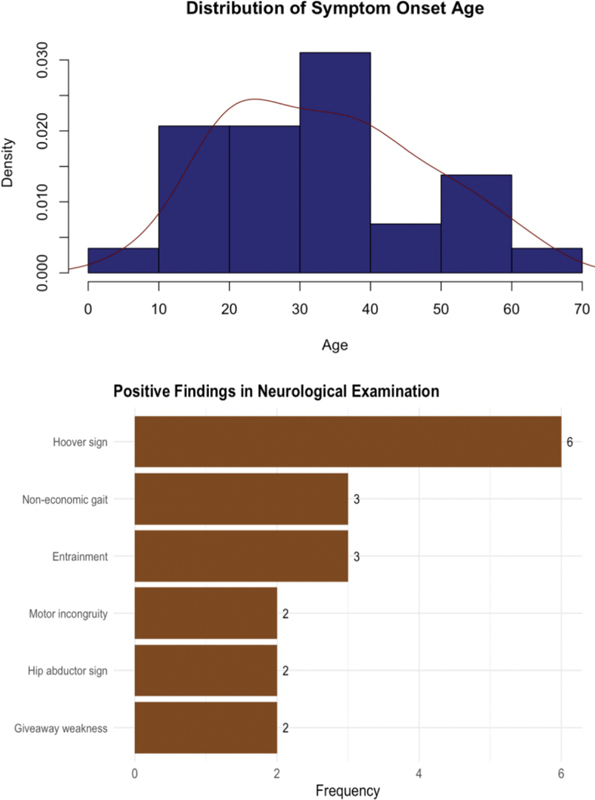
Age distribution (histogram) and frequency of positive neurological signs (bar chart) identified in the study population (n = 29). Positive signs include Hoover's sign, noneconomic gait, and tremor entrainment, among others.

**Figure 2:**
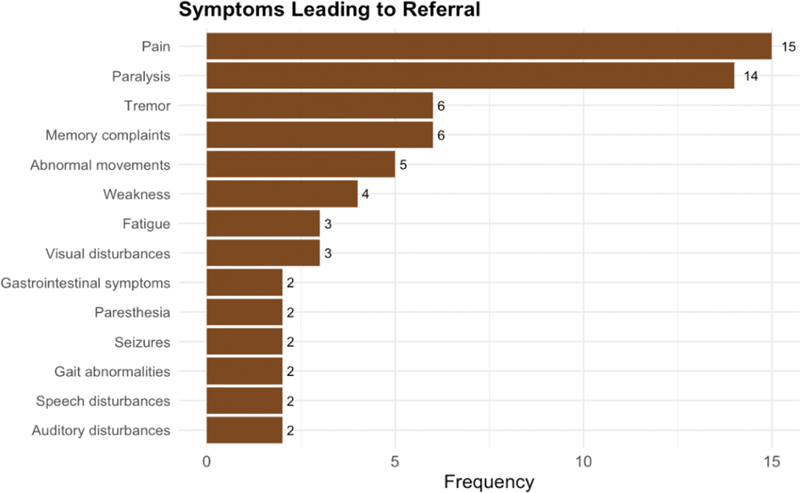
Distribution of FND clinical subtypes leading to referral to the SOMA outpatient unit. Categories include motor symptoms (paresis, tremor, dystonia, gait disorder), pain, PNES, sensory disturbances, and speech/voice alterations.

A total of 29 patients were included, 22 of whom (75.9%) were cisgender women and 7 (24.1%) were cisgender men. Mean age was 33.6 ± 13 years. The most common clinical presentations were pain (n = 15; 51.7%) and paresis (n = 14; 48.3%). Other FND subtypes present in the cohort included patients with PNES, combined motor and sensory presentations, and speech alterations. It should be noted that patients with isolated PNES or purely sensory/autonomic presentations were managed in parallel services and are not included in this sample. The most frequent positive neurological sign was Hoover's sign (n = 6; 20.7%), followed by non-economic gait (n = 3; 10.3%) and tremor entrainment (n = 3; 10.3%). Figure 1 displays the age distribution and frequency of positive neurological signs in the study population. Figure 2 shows the distribution of FND clinical subtypes responsible for referral to psychiatric treatment.

**Figure 3:**
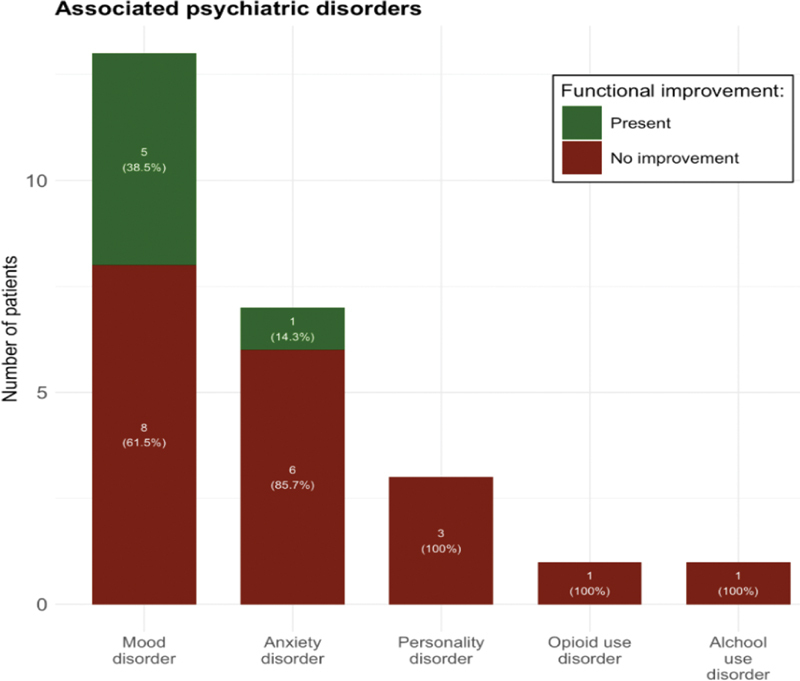
Distribution of psychiatric comorbidities (mood disorders, anxiety disorders, personality disorders, and no psychiatric diagnosis) and proportion of patients achieving functional improvement within each group.

Associated psychiatric disorders included mood disorders (n = 13; 44.8%), generalized anxiety disorder (n = 6; 20.7%), and personality disorders (n = 3; 10.3%). Psychiatric diagnoses were established by board-certified psychiatrists based on DSM-5 criteria, supported by structured clinical interview. Figure 3 shows the distribution of psychiatric comorbidities and the proportion of functional improvement within each group.

Over the course of treatment, 9 patients (31%) achieved functional improvement. In terms of specific treatment modalities received, 25 patients underwent group psychotherapy, 13 physiotherapy, 9 eutony, 7 individual psychotherapy, and 4 acupuncture. Several patients received more than one treatment modality simultaneously; each modality was analyzed as an independent predictor in the regression model.

### Functional improvement outcome

In the multivariable regression model, receipt of group psychotherapy was independently associated with functional improvement during follow-up ( *p* = 0.036). In contrast, patients who did not receive any therapeutic intervention were significantly less likely to improve ( *p* = 0.003).

Eutony had a significant association with functional improvement ( *p* < 0.05). Patients who received eutony had 5.68 times higher odds of experiencing functional improvement compared to those who did not (odds ratio [OR] = 5.68).

Regarding the association between mood disorders and outcome, patients without a mood disorder diagnosis had significantly lower odds of achieving functional improvement compared to those with (OR = 0.36; 95% confidence interval [CI]: 0.11–0.99; *p* = 0.048). In other words, the presence of a mood disorder comorbidity was associated with a nearly threefold increase in the odds of functional improvement. There was no statistically significant association between sex and functional improvement ( *p* > 0.05).

## DISCUSSION

The present study describes the clinical characteristics, psychiatric comorbidities, and therapeutic outcomes of a retrospective cohort of Brazilian patients with FND. Our findings reinforce the international literature on several central points but also highlight particular aspects that deserve emphasis.

To the best of our knowledge, this is the first Brazilian study to employ multivariable statistical modeling to identify clinical and therapeutic predictors of functional improvement in patients with FND. Additionally, we highlight the role of group psychotherapy and eutony as potentially impactful interventions, contributing to the international literature with original data from a Latin American clinical service.

### Demographic and clinical profile

The predominance of young women (75.9%; mean age: 33.6 years) is consistent with international studies showing a higher prevalence in women, especially between the third and fifth decades of life. 1 2 This pattern has been attributed to biopsychosocial factors, including vulnerabilities associated with early trauma and gender disparities in mental health. 4 Moreover, the most frequent symptoms in our sample—pain and functional paresis—align with findings from large cohorts, in which weakness, movement disorders, and somatosensory symptoms are the main reasons for seeking medical care. 3 4 20

### Positive diagnostic findings

Among positive signs, Hoover's was the most frequently observed. This is relevant as it underscores the importance of clinical diagnosis based on specific semiological findings, as advocated since the DSM-5 revision. 7 International studies emphasize that the use of these positive signs increases diagnostic confidence, reduces stigma, and enhances treatment adherence. 12 14 The presence of signs such as tremor entrainment and uneconomical gait in part of our sample also corroborates international descriptions. 21

### Therapeutic interventions

The analysis of functional outcomes revealed an improvement rate of 31%, similar to systematic reviews suggesting that only ⅓ of patients achieve partial or complete recovery in the medium term. 18 This finding confirms the generally unfavorable prognosis of FND, although early introduction of interventions may improve outcomes.

It is important to acknowledge that treatment allocation in our cohort was not randomized but followed a standardized clinical protocol, as described in the Methods section. The heterogeneity of treatment modalities reflects the real-world complexity of managing FND in a multidisciplinary setting. Many patients received more than one intervention simultaneously, which precludes definitive causal inference for individual treatments. The regression model controlled for multiple modalities as independent variables, but the limited sample size imposes constraints on interpretation. These findings should, therefore, be regarded as hypothesis-generating rather than confirmatory.

In our cohort, group psychotherapy was the factor most strongly associated with functional improvement. This resonates with international evidence highlighting the relevance of diagnostic education, collective psychoeducational support, and integrative psychotherapy in the management of FND. 1 6 15 The group setting may foster the exchange of experiences, reduce isolation, and reinforce the perception of the disorder's legitimacy, aspects recognized as fundamental for clinical evolution. 13

Another noteworthy finding was the positive association between eutony and functional improvement. Although scarcely explored in international studies, eutony shares principles with body-oriented approaches, such as functional physiotherapy and occupational therapy, already validated in international consensus recommendations. 15 16 21 Our study suggests that somatic reeducation practices may be particularly promising in the Brazilian context, broadening the range of integrative interventions for FND. Although large effect sizes were observed, these estimates should be interpreted cautiously given the small sample size and potential model instability.

### Psychiatric comorbidities

Mood and anxiety disorders were the most common comorbidities, consistent with studies reporting prevalence rates of up to 50 to 70% in FND populations. 16 17

All patients with identified psychiatric comorbidities received appropriate treatment along with their FND management. Patients with mood or anxiety disorders were treated with pharmacotherapy (antidepressants or anxiolytics, as clinically indicated) and/or individual or group psychotherapy. Personality disorder diagnoses prompted referral for specialized psychological follow-up. Although we did not model the specific effects of psychiatric treatment on FND outcomes due to sample size constraints, the comanagement of psychiatric comorbidities was a standard component of the multidisciplinary care provided at SOMA-IPq.

Interestingly, in our sample, the absence of mood disorders was associated with a lower likelihood of functional improvement. This finding may indicate that having a prior psychiatric diagnosis facilitates faster integration into therapeutic programs and reduces resistance to the functional diagnosis. Although this hypothesis requires confirmation in multicenter studies, it suggests that the relationship between psychiatric comorbidities and prognosis in FND may be more complex than previously described.

### Overall interpretation

Overall, the findings of this study align with international data: predominance in young women; 1 2 motor symptoms and pain as primary presentations; 3 4 20 positive signs as diagnostic markers, with Hoover's sign in particular; 7 12 14 limited prognosis, with improvement rates around ⅓; 18 and the importance of psychotherapy and multidisciplinary approaches. 1 6 15 16 17 21

Our study adds evidence that integrative body-oriented practices, such as eutony, may play a relevant role in the therapeutic context. Furthermore, it suggests that psychiatric comorbidities, rather than being merely risk factors, may in some cases facilitate therapeutic engagement and improve adherence.

### Limitations

The limitations of this study include its small sample size and retrospective design, which may restrict the generalizability of the findings. Secondly, as patients are still under follow-up, data on the long-term maintenance of functional improvement are not yet available. Furthermore, the main outcome, functionality, was not assessed by a validated quantitative instrument, which reduces consistency of the findings. The absence of randomization in treatment allocation and the co-occurrence of multiple interventions limit causal inference. Finally, the single-center design may restrict the external validity of the results.

### Perspectives

Future multicenter, prospective studies with larger samples are needed to confirm the effects of group psychotherapy and eutony on FND prognosis. Investigations into the mechanisms through which psychiatric comorbidities influence clinical evolution are also highly relevant. The development and validation of structured outcome measures for FND in the Brazilian and Latin American context represents an important research priority.
